# Glutenin from the Ancient Wheat Progenitor Is Intrinsically Allergenic as It Can Clinically Sensitize Mice for Systemic Anaphylaxis by Activating Th2 Immune Pathway

**DOI:** 10.3390/ijms25137324

**Published:** 2024-07-03

**Authors:** Rick Jorgensen, Tamil Selvan Arul Arasan, Maya Blanka Srkalovic, Chris Van Antwerp, Perry K. W. Ng, Venu Gangur

**Affiliations:** 1 Food Allergy and Immunology Laboratory, Department of Food Science and Human Nutrition, Michigan State University, East Lansing, MI 48823, USA; jorgen70@msu.edu (R.J.); arultami@msu.edu (T.S.A.A.); msrkalovic6397@gmail.com (M.B.S.); vanant29@msu.edu (C.V.A.); 2 Cereal Science Laboratory, Department of Food Science and Human Nutrition, Michigan State University, East Lansing, MI 48823, USA; ngp@msu.edu

**Keywords:** gluten allergy, ancient wheat, glutenin, life-threatening anaphylaxis, sensitization, Th2 pathway

## Abstract

Wheat allergy is a major type of food allergy with the potential for life-threatening anaphylactic reactions. Common wheat, *Triticum aestivum* (hexaploid, AABBDD genome), was developed using tetraploid wheat (AABB genome) and the ancient diploid wheat progenitor (DD genome)-*Aegilops tauschii*. The potential allergenicity of gluten from ancient diploid wheat is unknown. In this study, using a novel adjuvant-free gluten allergy mouse model, we tested the hypothesis that the glutenin extract from this ancient wheat progenitor will be intrinsically allergenic in this model. The ancient wheat was grown, and wheat berries were used to extract the glutenin for testing. A plant protein-free colony of Balb/c mice was established and used in this study. The intrinsic allergic sensitization potential of the glutenin was determined by measuring IgE response upon transdermal exposure without the use of an adjuvant. Clinical sensitization for eliciting systemic anaphylaxis (SA) was determined by quantifying the hypothermic shock response (HSR) and the mucosal mast cell response (MMCR) upon intraperitoneal injection. Glutenin extract elicited a robust and specific IgE response. Life-threatening SA associated and a significant MMCR were induced by the glutenin challenge. Furthermore, proteomic analysis of the spleen tissue revealed evidence of in vivo Th2 pathway activation. In addition, using a recently published fold-change analysis method, several immune markers positively and negatively associated with SA were identified. These results demonstrate for the first time that the glutenin from the ancient wheat progenitor is intrinsically allergenic, as it has the capacity to elicit clinical sensitization for anaphylaxis via activation of the Th2 pathway in vivo in mice.

## 1. Introduction

The identification of wheat as a prevalent instigator of immune-mediated food allergies has emerged as a significant global health concern for public health and food safety. As wheat has the potential for life-threatening anaphylaxis, this poses a substantial health and financial burden [1]. In a recent global meta-analysis of wheat allergy, it was found that 0.22% to 1.93% of individuals are affected by this condition [2]. The prevalence varies across different regions of the world, based on self-reported physician-diagnosed wheat allergy [2]. With no cure for wheat allergy at present, individuals are required to adhere to strict elimination diets, impacting their quality of life through an elevated risk for severe allergic reactions at any time [3].

Wheat proteins encompass both gluten and non-gluten components. The gluten fraction constitutes 80–85% of the overall wheat proteins, while the remaining 15–20% is comprised of the water-soluble and salt-soluble non-gluten fractions [4]. Gluten serves as storage proteins in wheat and other cereals like rye or barley, which are all cereals belonging to the *Poaceae* family [5]. The alcohol-soluble glutens, known as gliadins, are found as individual proteins that interact via hydrogen bonds and predominantly feature intramolecular disulfide bonds. In contrast, the acid-soluble glutens, known as glutenins are polymeric proteins that establish connections through both intermolecular and intramolecular disulfide bonds. Moreover, gliadins can also be linked to the glutenin network through intermolecular disulfide bonds [6]. Among the alcohol-soluble gluten proteins, ω-1, 2, 5 gliadins and α/β/γ-gliadins have been thoroughly characterized and are recognized for inducing allergic reactions in susceptible individuals [1]. Within the acid-soluble fraction, both high-molecular-weight (HMW) and low-molecular-weight (LMW) glutenin subunits are able to bind IgE antibodies, causing severe allergic reactions [1,7,8].

Wheat exhibits significant genetic diversity within both domesticated and wild species, spanning various ploidy levels. Durum wheat, *Triticum durum* (AABB genome), is a tetraploid wheat containing the A and B genomes. *Triticum aestivum* (AABBDD genome) is a hexaploid wheat containing the A, B, and D genomes, originating from two interspecific hybridization events between the tetraploid emmer wheat, *Triticum turgidum* (AABB genome), and the diploid wheat, *Aegilops tauschii* (DD genome), progenitors [9]. Most studies of gluten allergenicity have used tetraploid and hexaploid wheat, and in some cases, diploid Einkorn (AA genome) wheat [10,11,12,13,14,15]. However, the intrinsic allergenicity of glutens from the ancient wheat *Aegilops tauschii* (DD genome), which is not commercially available, is unknown. Elucidating the allergenicity potential of ancient wheats such as *Ae. tauschii* is critical for the following reasons: (i) the whole genome of *Ae. tauschii* has previously been sequenced [16], and it is proposed that this genetic information will be valuable to improve the genetics of modern wheat crops to enhance their quality and safety; and (ii) there are scientific discussions in the literature that ancient wheat species may have lower immunogenicity and allergenicity compared with the modern common wheat; however, direct evidence of the intrinsic allergenic potential of glutens from the ancient wheat is lacking [17].

Various animal models, including dogs, rats, and mice, have been employed to investigate gluten allergenicity [15,18,19,20,21,22,23]. These models may be classified into two general types based on the sensitization protocols: (i) the intraperitoneal administration of gluten plus adjuvants such as alum or Fruend’s adjuvant, to enhance the immune response to gluten so that marked IgE responses are initiated and (ii) the administration of gluten transdermally without an adjuvant to induce robust IgE responses. The former approach has been widely used. However, despite their utility and unique strengths, these adjuvant-based models cannot be used to evaluate the inherent allergenicity potential of glutens, because of the enhancement of non-specific immune stimulation due to the adjuvant’s action on innate immune cells as well as adaptive immune responses [24,25]. In contrast, the latter approach, which does not utilize adjuvants to elicit IgE responses, has the attractive capacity to decipher the inherent allergenicity potential of glutens.

Therefore, in this study, we utilized an adjuvant-free mouse model to investigate the hypothesis that the glutenin extract from this ancient wheat progenitor will be intrinsically allergenic in this model. The study encompassed four objectives: (i) to assess the sensitization (IgE antibody) and disease eliciting capacity of glutenin extract from the ancient wheat *Ae. tauschii*; (ii) to quantify systemic anaphylaxis-eliciting potentials of glutenin in this model as quantified by the hypothermic shock responses (HSRs) upon intraperitoneal injection; (iii) to quantify the mucosal mast cell degranulation responses (MMCRs) induced by intraperitoneal injection of the glutenin; and (iv) to map the spleen immune marker activation signatures associated with systemic anaphylaxis induced by the ancient wheat glutenin.

This study collectively demonstrates for the first time that the glutenin from the ancient wheat progenitor is intrinsically allergenic, as it has the capacity to elicit clinical sensitization for anaphylaxis via activation of the Th2 pathway in vivo in mice.

## 2. Results

### 2.1. Transdermal Exposure to Glutenin Extract from Aegilops tauschii Elicits Robust Specific-IgE Antibody Response in Balb/c Mice

**Figure 1:**
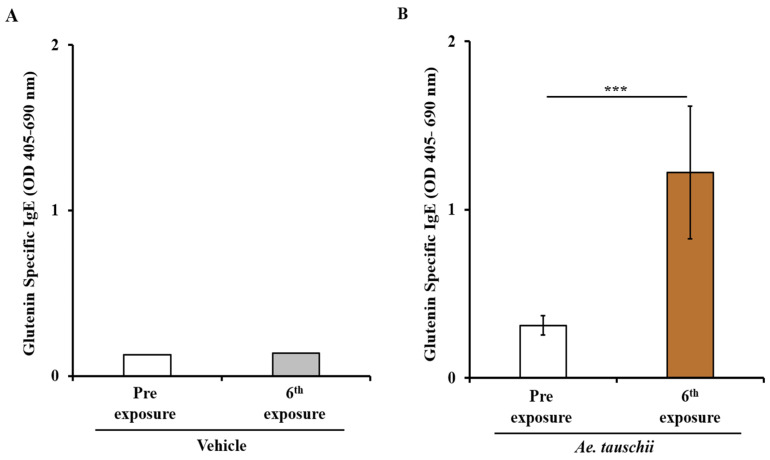
Transdermal exposure of Balb/c mice to glutenin extract from *Ae. tauschii* (genome DD) elicited robust and specific (s)IgE antibody responses. Mice were exposed to glutenin extract from *Ae. tauschii* or vehicle as described in Methods. Plasma collected before the 1st exposure (Pre) and after the 6th exposure (6R) was used in the measurement of sIgE levels (OD 405–690 nm), with a sample size of *n* = 10/group. (**A**) sIgE levels in control mice. (**B**) sIgE levels in sensitized mice. *** *p* < 0.001, Student’s *t*-test. *n*: number of mice; OD: optical density.

Balb/c female mice were grouped and exposed to transdermal application of glutenin extract from *Aegilops tauschii* (genome DD) or a vehicle, following a repeated weekly exposure regimen as outlined in the Methods Section. Blood samples collected before the initial exposure (pre) and after the sixth skin exposure (6R) were analyzed for specific (s)IgE levels. Significantly, there was a substantial elevation in sIgE antibody levels after transdermal exposure to the glutenin extract from *Ae. tauschii* compared to the vehicle control mice, as depicted in Figure 1A,B.

### 2.2. Systemic Challenge with Aegilops tauschii Glutenin Elicits Hypothermic Shock Responses in Skin-Sensitized Mice

**Figure 2:**
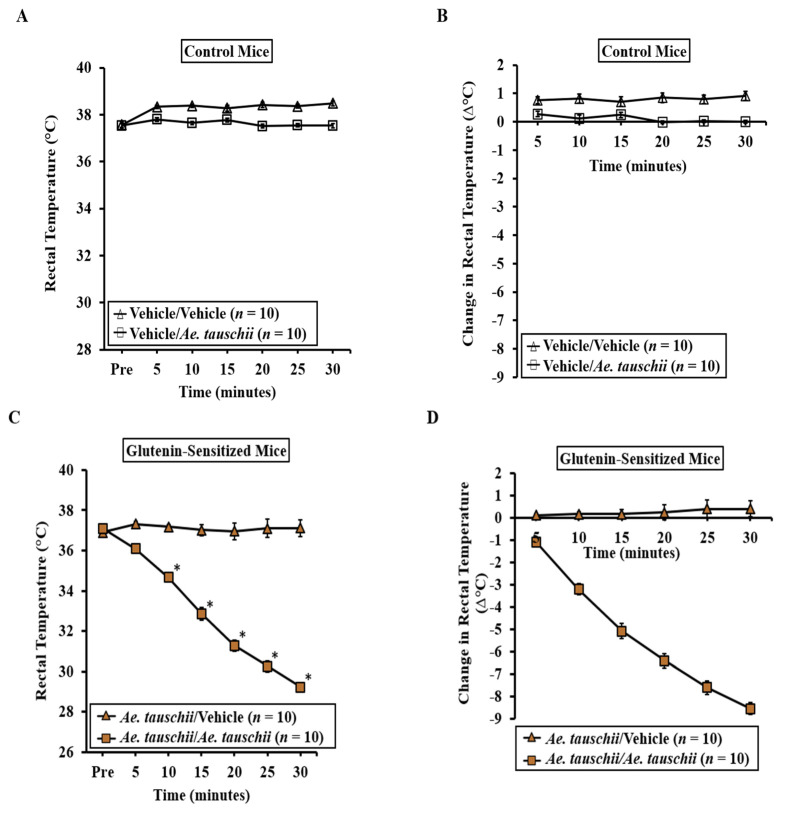
Transdermal exposure to glutenin from *Aegilops tauschii* is sufficient for eliciting clinical sensitization for systemic anaphylaxis in Balb/c mice. Mice were sensitized and systemically challenged with glutenin extract from *Ae. tauschii* or vehicle, as described in the Materials and Methods Section. *n* = 10/group. (**A**) Actual rectal temperature at indicated time points in control mice challenged with glutenin extract from *Ae. tauschii* or vehicle. (**B**) Change in rectal temperature at indicated time points in control mice challenged with glutenin or vehicle. (**C**) Actual rectal temperature at indicated time points in glutenin-sensitized mice challenged with glutenin or vehicle. (**D**) Change in rectal temperature at indicated time points in glutenin-sensitized mice challenged with glutenin or vehicle. *n*: number of mice; * student’s t-test *p* < 0.05.

Groups of skin-sensitized mice were employed to induce anaphylaxis through a systemic challenge with the *Ae. tauschii* glutenin extract (0.5 mg/mouse) or vehicle. Anaphylactic reactions were quantified using hypothermic shock reactions (HSRs) and monitored with rectal thermometry, as detailed in the Methods Section. No HSR was observed in control mice upon the vehicle (i.e., zero allergens) or glutenin extract challenge (Figure 2A,B). In contrast, acute HSRs were evident upon systemic allergen challenge in sensitized mice (Figure 2C,D). Significant HSRs were noted from 10 to 30 min post-systemic allergen challenge, as indicated by ANOVA analysis (*p* < 0.05).

### 2.3. Sensitized Balb/c Mice Exhibiting Systemic Anaphylaxis Have Strong Mucosal Mast Cell Response (MMCR) as Evidenced by Significant Elevation of MMCP-1 in the Blood

**Figure 3:**
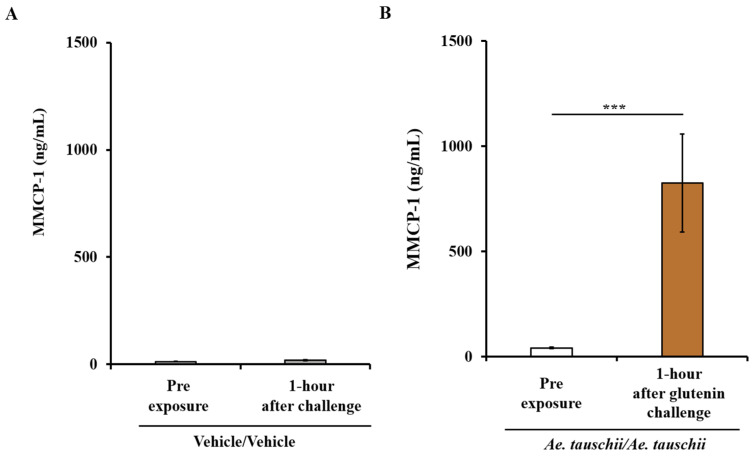
Systemic anaphylaxis elicited by the ancient wheat glutenin is associated with a robust mucosal mast cell response (MMCR) in Balb/c mice. Mice were sensitized and systemically challenged with glutenin or vehicle, as described in the Materials and Methods Section. Plasma levels of mucosal mast cell protease (MMCP)-1 (ng/mL) before and one hour after challenge were measured using ELISA. *n* = 10/group. (**A**). MMCP-1 levels in control mice challenged with the vehicle. (**B**) MMCP-1 levels in allergic mice challenged with glutenin from *Ae. tauschii*. *** *p* < 0.05, Student’s *t*-test; *n*: number of mice.

Figure 3A,B illustrate the MMCP-1 responses in both control mice and allergic mice. It is evident that the systemic challenge with glutenin, as opposed to vehicle, results in a significant increase in MMCP-1 levels in the blood, as depicted in Figure 3A,B.

### 2.4. Proteomic Analysis and Mapping the Differentially Expressed Immune Marker Signatures in the Spleen of Mice Undergoing Systemic Anaphylaxis Elicited by the Ancient Wheat Glutenin

**Figure 4:**
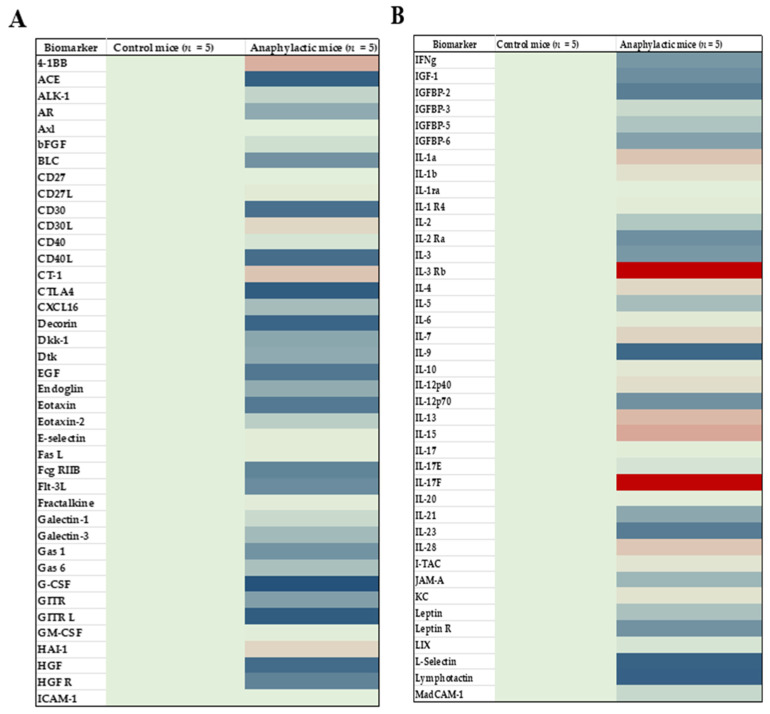

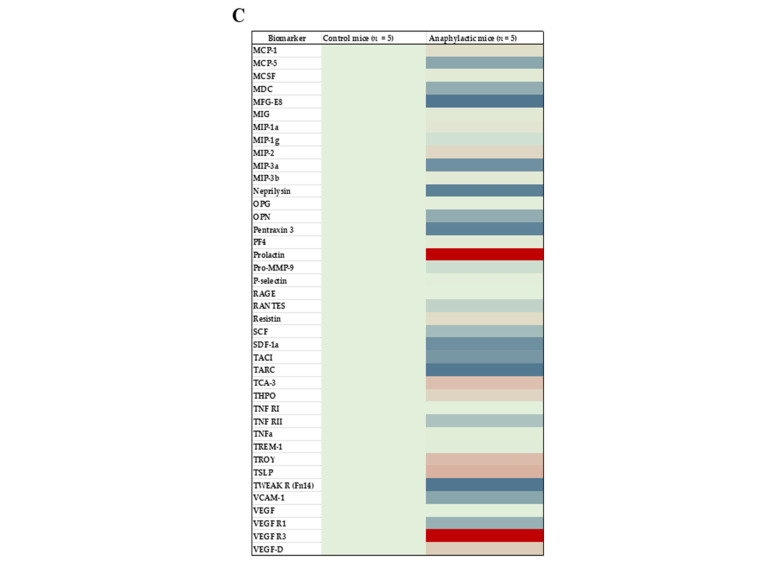
Heat map analysis of 120 spleen immune biomarkers in ancient wheat glutenin-induced systemic anaphylaxis. Using spleen extracts from control mice and anaphylactic mice, a proteomic microarray analysis was conducted using Ray-Biotech system cytokine panels, (**A**) CYT-4, (**B**) CYT-5, and (**C**) CYT-6, as described in the Methods Section. Background levels of immune biomarkers are shown in green. Upregulated biomarkers are shown in red and downregulated biomarkers are shown in blue.

Using biomarker data obtained from the proteomic analysis, heat maps were created to identify changes in biomarker protein expression during systemic anaphylaxis elicited by the ancient wheat glutenin; several biomarkers were affected by systemic anaphylaxis (Figure 4A–C). Among the impacted biomarkers, 34 markers including the prototypic Th2 markers (IL-4, IL-13, and TSLP) were significantly elevated, and 55 were significantly reduced (Student’s *t*-test, two-tailed, *p* < 0.05) in anaphylactic mice.

**Figure 5:**
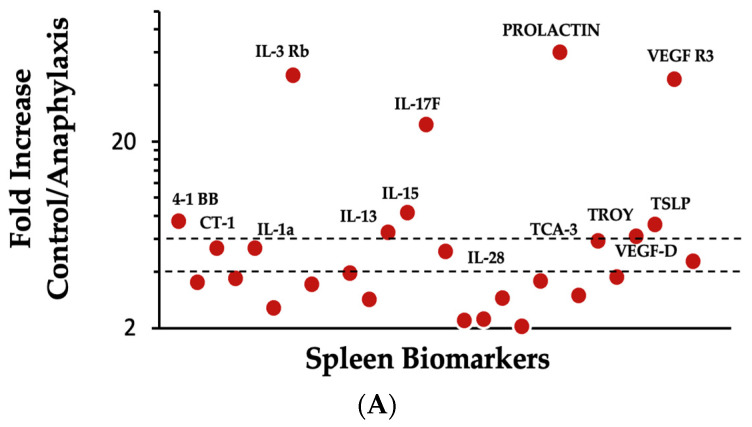

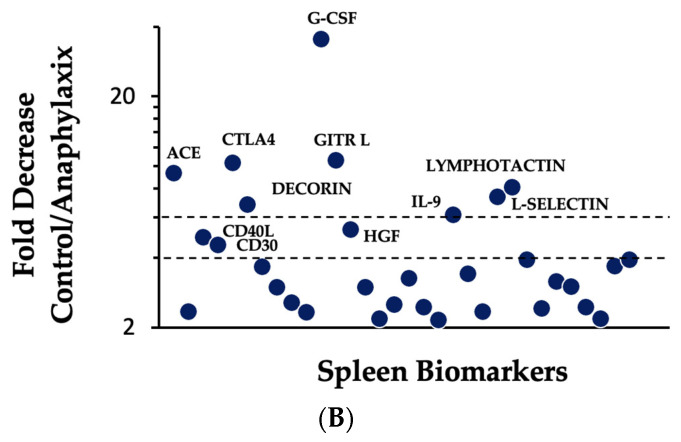
Identification and mapping the differentially expressed immune biomarkers in the spleen of mice showing anaphylaxis elicited by the ancient wheat glutenin. (**A**) Shows the relative protein expression map of the immune biomarkers that are significantly elevated (2-fold or higher, *p* < 0.05) during glutenin-induced anaphylactic mice. (**B**) Shows the relative protein expression map of the immune biomarkers that are significantly decreased (2-fold or lower, *p* < 0.05) during glutenin-induced anaphylaxis. The dotted lines indicate 4-fold and 6-fold changes in protein expression. Immune biomarkers that show 4-fold or higher changes are identified with names.

We have previously published a method to classify anaphylaxis-associated biomarkers [11]. In this method, makers are classified into four categories based on the fold-change in protein expression as follows: low importance (up to 1.9-fold change), medium importance (2–3.9-fold change), high importance (4–5.9-fold change), and critical importance (6 and above-fold change). The following 14 immune biomarkers were substantially elevated in anaphylaxis: VEGF-D, IL-28, CT-1, IL-1a, and TCA3 (high importance) and TROY, IL-13, TSLP, 4-1BB, IL-15, IL-17F, VEGF-R3, IL-3Rb, and Prolactin (critical importance) (Figure 5A). In contrast, the following 10 immune biomarkers were markedly reduced during anaphylaxis: CD40L, HGF (high importance), IL-9, Decorin, L-selectin, lymphotactin, ACE, CTLA4, GITR L, and G-CSF (critical importance) (Figure 5B).

## 3. Discussion

*Aegilops tauschii* is the ancient progenitor with the DD genome that enabled the creation of the modern common wheat (hexaploid bread wheat, AABBDD genome) [26]. This ancient wheat is not currently cultivated, and therefore, it is not commercially available. We recently grew the ancient wheat progenitor *Ae. tauschii* for research purposes at Michigan State University [27,28]. The potential allergenicity of gluten from this ancient wheat is unknown. Therefore, this study was undertaken to test the hypothesis that the glutenin extract from this ancient wheat progenitor will be intrinsically allergenic in an adjuvant-free mouse model. Collectively, our data uphold this hypothesis.

There are seven novel findings in this study: (i) the glutenin extract from *Aegilops tauschii* is intrinsically allergenic because chronic dermal exposure to this protein extract without an adjuvant induces a significant specific IgE antibody response in Balb/c mice; (ii) skin exposure to this glutenin is capable of clinically sensitizing mice for life-threatening anaphylaxis because dramatic hypothermic shock responses (HSRs) were observed upon systemic challenge with glutenin in skin-sensitized mice; (iii) systemic anaphylaxis elicited by the ancient wheat glutenin was associated with the elevation of mucosal mast cell protease-1 (MMCP-1) in the blood, suggesting a mucosal mast cell degranulation response (MMCR) occurring in the gut; (iv) systemic anaphylaxis is associated with a significant elevation of prototypic Th2 immune markers, IL-4, IL-13, and TSLP; (v) systemic anaphylaxis is associated with a signature set of immune markers that are dramatically elevated (4-fold or higher compared to the control mice); (vi) systemic anaphylaxis is also associated with a signature set of immune markers that are dramatically downregulated (4-fold or lower compared to the control mice); and (vii) collectively, we demonstrate the intrinsic allergenic potential of the glutenin protein extract from the ancient wheat progenitor for the first time.

We chose to utilize a recently described adjuvant-free mouse model to test the allergenic potential of glutenin from *Ae. tauschii* for the following reasons: (i) previously, this model has been validated using glutenin from *Triticum aestivum* (AABBDD genome) [11,12]; therefore, the genetic contribution of the ancient wheat progenitor of the DD genome to the glutenin allergenicity of modern wheat could be elucidated; (ii) as opposed to adjuvant-based models, because this model does not use an adjuvant, it provides a unique opportunity to evaluate the inherent allergenicity potential of glutenin from the ancient wheat progenitor; (iii) this model is also relatively more humane compared to the other skin sensitization models, such tape-stripping or sandpaper-rubbing methods to remove upper layer of skin to enhance allergen penetration via the skin; (iv) one sensitization marker (specific IgE in the blood) and two quantitative markers of disease elicitation—HSR by rectal thermometry and MMCR by measuring the elevation of MMCP-1 in the blood—have been validated previously, thus providing an opportunity to objectively determine the intrinsic allergenicity potential of glutenin from the ancient wheat progenitor.

In this study, we chose to evaluate the allergenicity potential of the ancestral *Aegilops tauschii* wheat for the following reasons: (i) *Ae. tauschii* is the ancestral uncultivated wild progenitor that provided the DD genome for the modern common bread wheat (*Triticum aestivum*, AABBDD genome); however, the allergenicity of gluten from this ancient wheat progenitor is unknown [26,29]; (ii) at our university, we had unique access to *Ae. tauschii* seeds and experimentally cultivated the *Ae. tauschii* crop; we also previously reported the allergenicity of non-gluten protein from this ancient wheat [28,30]; (iii) previously, the whole genome of *Ae. tauschii* has been deciphered with the hope of creating improved varieties of modern wheat that are safer and more nutritious [16]; and (iv) there are scientific discussions in the literature debating the potential lower allergenicity of ancient wheats compared to that of the modern widely consumed wheats; however, the results are still inconclusive and more evidence is needed; therefore, the results from this study will now advance the science by filling this gap in the scientific knowledge on the allergenicity potential of gluten from this ancient wheat progenitor [17].

The mechanism of wheat glutenin allergy is thought to occur in two phases [31]. The first phase occurs when genetically predisposed individuals are exposed to glutenin through the skin or other routes. This exposure leads to the production of glutenin-specific IgE antibodies [32,33,34]. These antibodies then attach to mast cells and basophils via high-affinity IgE receptors, resulting in a sensitized immune state. The second phase occurs when a sensitized individual is re-exposed to glutenin. The glutenin allergens bind to the IgE-loaded mast cells and basophils, triggering the release of chemical mediators that cause clinical disease, including systemic anaphylaxis [4]. Therefore, in order to evaluate the sensitization potential of the ancient wheat glutenin, we measured specific IgE antibodies, using an ultra-sensitive ELISA-based method that we have reported previously [11,12,28]. Our data demonstrate very clearly that the glutenin from the ancient wheat *Aegilops tauschii* has the inherent capacity to elicit sensitization via skin exposure.

The hypothermic shock response (HSR) represents the consequence of anaphylaxis on neurological and cardiovascular functions involved in thermoregulation [35]. The HSR, measured via rectal thermometry, is a widely used method to quantify the severity of anaphylaxis in mouse models of food allergy, as it is considered a proxy marker of cardiac output which is adversely affected in food-induced systemic anaphylactic reactions [11,12,28,36,37,38,39,40]. Our data demonstrate that the ancient wheat glutenin can elicit a significant HSR, suggesting its capacity to elicit systemic disease in mice.

Mucosal mast cell protease-1 (MMCP-1) was previously reported as a specific biomarker of IgE-antibody-mediated but not IgG-antibody-mediated systemic anaphylaxis in food allergy mouse models [41]. Since HSRs may reflect both IgE- and IgG1-mediated systemic anaphylaxis in mice, we chose to use MMCP-1 as an additional biomarker to specifically determine whether glutenin from *Aegilops tauschii* can elicit life-threatening anaphylaxis via the IgE and mucosal mast cell pathway. Furthermore, the only source of MMCP-1 in mice is the mucosal mast cells. The gastrointestinal tract harbors mucosal mast cells containing MMCP-1 in the granules, which are a distinctive protein found exclusively in mucosal mast cells and absent in connective tissue mast cells [42]. These gut mucosal mast cells possess receptors for IgE antibodies. Following sensitization to glutenin, specific IgE antibodies attach to mucosal mast cells through high-affinity IgE antibody receptors (FcϵRI). In the pre-challenge samples, blood exhibits minimal background levels of MMCP-1, since the mucosal mast cells remain inactive. However, upon challenge with glutenin, the glutenin binds to the IgE antibodies on the gut mast cells, triggering the activation and degranulation of the mucosal mast cell. This process releases MMCP-1 into the systemic circulation. Therefore, the elevated MMCP-1 levels upon allergen challenge indicate IgE-antibody-mediated anaphylaxis in the gut, which simulates the natural IgE-mediated reactions that occur in glutenin allergy upon ingestion of glutenin-containing products. Our data demonstrate that the ancient wheat progenitor glutenin can activate the classical IgE/mucosal mast cell activation and degranulation pathway causing systemic anaphylaxis.

To elucidate the immune mechanisms associated with the systemic anaphylaxis elicited by the ancient wheat glutenin, we conducted a proteomic analysis of the spleen, which is an organ that is representative of the immune system. We have previously published a methodology to study biomarker fold changes in anaphylaxis [11]. Using this method, we mapped differentially expressed markers and identified five markers belonging to the high-importance category and nine belonging to the critical-importance category, both of which were positively linked to anaphylaxis as follows: VEGF-D, IL-28, CT-1, IL-1a, and TCA3 (high importance) and TROY, IL-13, TSLP, 4-1BB, IL-15, IL-17F, VEGF-R3, IL-3Rb, and Prolactin (critical importance). It is noteworthy that these markers have been previously shown to be important in various models of inflammation, allergy, anaphylaxis, and celiac disease [11,43,44,45,46]. Notably, along with IL-4, IL-13, and TSLP, critical cytokines are also implicated in allergenicity responses in general, and their positive association in this model provides a molecular mechanistic model to explain the intrinsic allergenicity of the glutenin from the ancient wheat progenitor [47]. Furthermore, VEGR-R3, a key molecule associated with vascular permeability, has been previously linked to anaphylaxis elicited by glutenin from the modern common wheat (*Triticum aestivum*) [11]. The cytokine IL-15 has been linked to gluten-induced celiac disease previously in mice and in humans [48]. All other markers identified are novel positive associations with glutenin-induced anaphylaxis.

We noted that many immune markers were markedly downregulated during anaphylaxis. Among them, two markers belonged to the high-importance category and eight belonged to the critical-importance category as follows: CD40L and HGF (high importance) and IL-9, Decorin, L-selectin, lymphotactin, ACE, CTLA4, GITR L, and G-CSF (critical importance). These immune markers are immune regulators that include cytokines, chemokines, cell surface immune regulators, and immune cell growth factors. The negative association with anaphylaxis may suggest potential protective effects of these molecules in glutenin-induced anaphylaxis for the first time; clearly, follow-up studies are needed to evaluate this possibility.

There are suggestions that the ancient wheat grains may be more tolerable for individuals with wheat allergies compared to the modern wheat varieties [17]. This hypothesis was developed to explain why wheat allergies and other immune-mediated reactions to wheat have been increasing in recent decades. This is an area of great interest for scientists that has received very limited investigation [17]. Therefore, the present research was undertaken to evaluate this hypothesis using the ancient *Ae. tauschii* wheat progenitor. Our results clearly demonstrate that the ancient wheat glutenin is indeed highly allergenic in this model. We would like to caution that the results from this study may or may not be applicable to human situations because this is a mouse model study. Future investigation is needed to confirm the findings from this study in human subjects with wheat allergies. For example, *Ae. tauschii* glutenin samples can be tested by ELISA/RAST, skin prick testing can be used to evaluate sensitization capacity, and oral challenge tests can be conducted to evaluate the anaphylaxis-eliciting potential in wheat-allergic humans.

In summary, the results from this study collectively demonstrate for the first time that the glutenin from the ancient wheat progenitor is intrinsically allergenic, as it has the capacity to elicit clinical sensitization for anaphylaxis via activation of the Th2 pathway in vivo in mice.

## 4. Materials and Methods

### 4.1. Chemicals and Reagents

Biotin-conjugated antibodies specific to mouse IgE were obtained from BD Bio-Sciences (San Jose, CA, USA). The p-nitrophenyl phosphate compound was sourced from Sigma (St. Louis, MO, USA), and Streptavidin alkaline phosphatase was acquired from Jackson ImmunoResearch (West Grove, PA, USA). Folin reagent was obtained from Bio-Rad (Hercules, CA, USA). Specific reagents, including the IgE Mouse Uncoated ELISA Kit with Plates, Streptavidin-HRP, TMB substrate, MCPT-1 (mMCP-1) Mouse Uncoated ELISA Kit with Plates, Avidin-HRP, and TMB substrate, were procured from Invitrogen (Waltham, MA, USA). The Tissue Protein Extraction Reagent (T-PERTM), a proprietary detergent with a composition of 25 mM bicine and 150 mM sodium chloride at pH 7.6, was obtained from ThermoFisher Scientific (Waltham, MA, USA). For protease inhibition, a cocktail of serine, cysteine, and acid proteases, along with aminopeptidases, was acquired from Sigma-Aldrich (St. Louis, MO, USA).

### 4.2. Mice Breeding and Establishment of a Plant-Protein-Free Mouse Colony

Breeder pairs of adult Balb/c mice were procured from The Jackson Laboratory (Bar Harbor, ME, USA). Upon their arrival, the mice were introduced to a stringent plant-protein-free diet (AIN-93G, Envigo, Madison, MI, USA). After a one-week acclimation period, conventional breeding methods were employed to initiate reproduction. Female mice aged 6–8 weeks from the litters were specifically chosen for this study. Throughout the entire research period, all mice were consistently maintained on a strict plant-protein-free diet (AIN-93G). All animal procedures strictly adhered to the guidelines established by Michigan State University.

### 4.3. Preparation of Glutenin Extract from the Ancient Ae. tauschii Wheat

The ancient *Ae. tauschii* was grown at Michigan State University as described previously [28]. Protein extraction from the ancient wheat progenitor flour targeted the isolation of acid-soluble wheat glutenin using the modified Osborne sequential extraction method [49]. Briefly, a mixture of flour and filter-sterilized 0.5 M NaCl (1:10, *m*/*v*) underwent continuous agitation for 2 h, followed by centrifugation at 20,000× *g* for 30 min. The resulting pellets were preserved for alcohol extraction. The salt-insoluble pellets were then mixed in a 1:10 ratio with 70% ethanol for 2 h and centrifuged at 20,000× *g* for 15 min, yielding the alcohol-soluble gluten extract supernatant and alcohol-insoluble pellets. The latter was saved for acid extraction. The alcohol-insoluble pellets were combined in a 1:4 ratio with 0.05 M acetic acid for two hours and centrifuged at 20,000× *g* for 15 min. The resulting supernatants from both extractions were frozen at −70 °C overnight and subjected to freeze-drying the next day. The lyophilized alcohol-soluble gluten extract was reconstituted using 70% ethanol, and the acid-soluble gluten extract was reconstituted using 0.05 M acetic acid to achieve a concentration of 1 mg protein per 100 µL for topical application. For challenges involving intraperitoneal (IP) injections, the glutenin was reconstituted with phosphate-buffered saline (PBS) to attain concentrations of 0.5 mg/mouse. Protein content was quantified using the LECO total combustion method from LECO (St. Joseph, MI, USA). SDS-PAGE testing was conducted to assess protein quality.

### 4.4. Skin Sensitization, Bleeding, and Plasma Sample Preparation

Female adult Balb/c mice were utilized for experimental purposes. To facilitate the procedures, the hair on the mice’s rumps was bilaterally removed using a Philips hair clipper (Amsterdam, The Netherlands). The wheat gluten extracts were administered onto the rump at a dosage of 1 mg per mouse in 100 µL, alternately using a vehicle solution of 0.05 M acetic acid. Following application, the treated area was covered with a non-latex bandage sourced from Johnson & Johnson (New Brunswick, NJ, USA) and left in place for one day. This process was repeated on a weekly basis, occurring nine times over a span of nine weeks. Blood samples were collected from the saphenous vein before the initial exposure and after the sixth exposure. The blood was drawn into tubes coated with the anticoagulant lithium heparin (Sarstedt Inc MicrovetteCB 300 LH, Nümbrecht, Germany). The collected blood was subsequently subjected to centrifugation to isolate plasma, which was then stored individually at −70 °C until required for subsequent testing of (s)IgE.

### 4.5. Elicitation of Systemic Anaphylaxis

Two weeks after the final cutaneous exposure to glutenin or the vehicle, the mice underwent an intraperitoneal (IP) injection. This injection involved either 0.5 mg of glutenin in PBS or the vehicle (phosphate-buffered saline, PBS).

### 4.6. Determination of Hypothermic Shock Responses

Rectal temperature (°C) measurements were recorded both before the challenge and at 5 min intervals following the challenge, up to a 30 min duration. A rectal thermometer (DIGI-SENSE, Boston, MA, USA) was used for these measurements. The recorded values included the specific temperatures and the corresponding differences (∆°C) compared to the pre-challenge temperatures for each individual mouse. These recorded data points were subsequently utilized for further analyses.

### 4.7. Measurement of Specific IgE Antibody Levels

Ancient wheat progenitor glutenin-specific (s)IgE antibody levels were quantified using a highly sensitive ELISA method, as previously detailed with certain modifications [11,12,50,51,52]. Initially, 96-well Corning 3369 plates were coated with glutenin and subsequently blocked using a 5% gelatin solution. After a thorough washing step, plasma samples were introduced onto the plate. Further washing ensued, followed by the addition of a biotin-conjugated anti-mouse IgE antibody. Subsequent washes were performed before introducing streptavidin alkaline phosphatase, and eventually, p-nitrophenyl phosphate was added to enable quantification, following established methodologies [11,12,51,52].

### 4.8. Quantification of Mucosal Mast Cell Protease-1 (MMCP-1) Level

One hour post-challenge, blood samples were obtained and employed to measure the levels of mucosal mast cell protease-1 (MMCP-1) in the plasma. The quantification was performed using an ELISA-based method developed by Invitrogen, following established procedures. In detail, 96-well Corning Costar 9018 plates were initially coated with a capture antibody (anti-mouse MMCP-1). Subsequently, both samples and standards (recombinant mouse MMCP-1) were added to the plate. A biotin-conjugated anti-mouse MMCP-1 antibody was then introduced as the secondary antibody. Detection was achieved using an avidin-HRP/TMB substrate system. It is noteworthy that the assay has a limit of detection set at 120 pg/mL, and the range of standards covered from 120 to 15,000 pg/mL. Testing was conducted in quadruplicate for each individual mouse’s plasma.

### 4.9. Proteomic Analysis of Spleen Immune Markers

Mice were euthanized one hour following the challenge, and their spleens were collected and snap-frozen in liquid nitrogen and stored at −70 °C. The tissue protein extraction process was followed by proteomic analysis using the Quantibody microarray (CYT-4, -5, and -6, with 120 marker panels involved in inflammation, immune regulation, and hypersensitivity) method and fold-change analysis of protein expression, as described previously [11].

## 5. Conclusions

These results demonstrate for the first time that glutenin from ancient *tauschii* wheat has the inherent potential to elicit skin sensitization and clinical sensitization for life-threatening anaphylaxis in an adjuvant-free mouse model. The long-term goal of our research project is to advance fundamental knowledge on the intrinsic allergenicity of various wheat species and wheat varieties to inform the development of potentially hypo/non-allergenic wheats suitable for wheat-allergic subjects. Toward this end, here we have characterized the intrinsic allergenicity of an ancient wheat, *Ae. tauschii* (genome DD), which was used to develop the modern common wheat (*Triticum aestivum* genome AABBDD). Previously, we have described the intrinsic allergenicity of glutenins from common wheat (genome AABBDD) [11]. Our studies provide the baseline intrinsic allergenicity of glutenins from ancient wheat. Therefore, future gene editing, modification, and deletion can be attempted on the ancient wheat to create hypoallergenic ancient wheat, where the currently described baseline allergenicity profile of the ancient wheat would serve as the comparative standard. Once validated using the allergenicity readouts described in this study, the hypoallergenic genetically altered ancient wheat could be used to create novel hexaploid wheats with an altered DD genome, which in principle, should provide a hypoallergenic novel hexaploid wheat (AABB″DD″ genome with altered hypoallergenic DD genome).

## Data Availability

The original contributions presented in the study are included in the article, further inquiries can be directed to the corresponding author.
